# Zinc oxide nanoparticles mediate bacterial toxicity in Mueller-Hinton Broth via Zn^2^^+^

**DOI:** 10.3389/fmicb.2024.1394078

**Published:** 2024-04-22

**Authors:** Alexander J. Caron, Iman J. Ali, Michael J. Delgado, Dustin Johnson, John M. Reeks, Yuri M. Strzhemechny, Shauna M. McGillivray

**Affiliations:** ^1^Department of Biology, Texas Christian University, Fort Worth, TX, United States; ^2^Department of Physics and Astronomy, Texas Christian University, Fort Worth, TX, United States

**Keywords:** metal oxides, zinc oxide, zinc toxicity, nanoparticles, nanoantibiotics, *Staphylococcus aureus*

## Abstract

As antibiotic resistance increases and antibiotic development dwindles, new antimicrobial agents are needed. Recent advances in nanoscale engineering have increased interest in metal oxide nanoparticles, particularly zinc oxide nanoparticles, as antimicrobial agents. Zinc oxide nanoparticles are promising due to their broad-spectrum antibacterial activity and low production cost. Despite many studies demonstrating the effectiveness of zinc oxide nanoparticles, the antibacterial mechanism is still unknown. Previous work has implicated the role of reactive oxygen species such as hydrogen peroxide, physical damage of the cell envelope, and/or release of toxic Zn^2+^ ions as possible mechanisms of action. To evaluate the role of these proposed methods, we assessed the susceptibility of *S. aureus* mutant strains, Δ*katA* and Δ*mprF*, to zinc oxide nanoparticles of approximately 50 nm in size. These assays demonstrated that hydrogen peroxide and electrostatic interactions are not crucial for mediating zinc oxide nanoparticle toxicity. Instead, we found that Zn^2+^ accumulates in Mueller-Hinton Broth over time and that removal of Zn^2+^ through chelation reverses this toxicity. Furthermore, we found that the physical separation of zinc oxide nanoparticles and bacterial cells using a semi-permeable membrane still allows for growth inhibition. We concluded that soluble Zn^2+^ is the primary mechanism by which zinc oxide nanoparticles mediate toxicity in Mueller-Hinton Broth. Future work investigating how factors such as particle morphology (e.g., size, polarity, surface defects) and media contribute to Zn^2+^ dissolution could allow for the synthesis of zinc oxide nanoparticles that possess chemical and morphological properties best suited for antibacterial efficacy.

## Introduction

There is an antibiotic crisis looming due to the rise of antibiotic-resistant infections coupled with decreased development of new antimicrobials (Fischbach and Walsh, 2009; Tacconelli et al., 2018). Metal oxide nanoparticles (NPs) are an unconventional yet promising antimicrobial therapy that could help address the ongoing rise of multidrug-resistant infections (Modi et al., 2022). NPs range from 1 to 100 nanometers and their small size results in a high surface-to-volume ratio which in turn impacts their physicochemical properties (Sirelkhatim et al., 2015; Modi et al., 2022). Zinc oxide (ZnO) NPs are particularly promising due to their antibacterial activity, biocompatibility, and low cost (Sirelkhatim et al., 2015; Lallo Da Silva et al., 2019; Gudkov et al., 2021). They have broad-spectrum activity including against multi-drug resistant pathogens such as methicillin-resistant *Staphylococcus aureus* (MRSA) (Kadiyala et al., 2018; de Lucas-Gil et al., 2019; Ali et al., 2021)*, Escherichia coli* (Preeti et al., 2020), *Acinetobacter baumannii* (Tiwari et al., 2018) *Klebsiella pneumonia* (de Lucas-Gil et al., 2019), and *Mycobacterium tuberculosis* (Heidary et al., 2019). Additionally, ZnO NPs contain properties, such as resistance to high heat and pressure and minimal production of harmful by-products, that make them amenable to applications in water purification, meat packaging, food canning, and coatings on medical implants (Li et al., 2008; Sirelkhatim et al., 2015; Akbar et al., 2019; Smaoui et al., 2023). Their long-term stability compared to traditional organic antibiotics also imparts potential utilization in remote regions of the world that do not have access to electricity or refrigeration (Modi et al., 2022).

Despite a large body of literature showing the effectiveness of ZnO NPs, the antibacterial mechanism of ZnO NPs is still unclear. Elucidating the mechanism may allow the design of more effective ZnO NPs as altering physical properties such as size, shape, polarity, and/or surface properties can change their electrochemical and thus antimicrobial properties (Lallo Da Silva et al., 2019; Gudkov et al., 2021). There are three prevailing theories as to the mechanism of ZnO antimicrobial activity. The first is production of reactive oxygen species (ROS), and in particular hydrogen peroxide (H_2_O_2_) (Xu et al., 2013; Sirelkhatim et al., 2015; Siddiqi et al., 2018; Tiwari et al., 2018; Lallo Da Silva et al., 2019; Gudkov et al., 2021). The second is physical damage due to direct interactions between particles and the cell that leads to membrane disruption, loss of cytoplasmic contents, and lysis (Feris et al., 2010; Sirelkhatim et al., 2015; Joe et al., 2017; Siddiqi et al., 2018; Akbar et al., 2019; Lallo Da Silva et al., 2019; Gudkov et al., 2021). The final is release of toxic Zn^2+^ ions from the ZnO NP surface that causes mis-metalation of enzymes and disruption of ionic homeostasis (Pasquet et al., 2014; Sirelkhatim et al., 2015; Siddiqi et al., 2018; de Lucas-Gil et al., 2019; Lallo Da Silva et al., 2019; Song et al., 2020; Gudkov et al., 2021).

We chose to investigate ZnO NP-mediated bacterial toxicity using *S. aureus* due to its clinical relevance and previous work showing its susceptibility, including MRSA strains, to ZnO NPs (Kadiyala et al., 2018; de Lucas-Gil et al., 2019; Ali et al., 2021). In addition to being one of the major causes of skin and soft tissue infections in the US (DeLeo and Chambers, 2009), MRSA is classified as a serious threat by the Centers for Disease Control (2019) and a high-priority pathogen by the World Health Organization (2017). The virulence of *S. aureus* has also been well-studied and many genetic mutants exist that can be used to test resistance mechanisms (Cheung et al., 2021). Using isogenic mutants that demonstrate altered susceptibility to known toxicity mechanisms, we found that neither H_2_O_2_ nor electrostatic interactions mediate ZnO NP toxicity in Mueller-Hinton Broth (MHB). Rather ZnO NPs inhibited bacterial growth in a contact-independent manner via soluble Zn^2+^ ions.

## Methods

### Bacterial strains and reagents

*S. aureus* strains (Newman or SA113) were grown in MHB (Hardy Diagnostics) at 37°C under aerobic (shaking) conditions unless otherwise noted. *S. aureus* Δ*katA* and Δ*mprF*, were created previously (Peschel et al., 2001; Park et al., 2008) with Δ*katA* from the Newman parental strain and Δ*mprF* from SA113. ZnO NPs were purchased from Sigma-Aldrich (catalog no. 544906) and the specific lots used in this study were MKCG5504 and MKBD9523. Confirmation of the predominant surface morphology of ZnO crystals was done by scanning electron microscopy (SEM) utilizing a JEOL FE-SEM instrument (JEOL, Peabody, MA, USA) at an operating voltage of 15 kV and a probe current of 9.6 A. Length calculations were determined by employing the ImageJ software (version 1.51, NIH, USA) on the SEM images. All other antimicrobials (H_2_O_2_, daptomycin, ZnCl_2_, and MgCl_2_) were also purchased from Sigma-Aldrich.

### MIC assays

Cultures were grown to an optical density (OD) of 0.4 at 600 nm then diluted 1:200 to approximately 1×10^6^ cfu/ml for all assays. For ZnO NP assays, bacteria were added to microcentrifuge tubes with the indicated concentrations of ZnO NPs in a final volume of 400 μL. The microcentrifuge tubes were continuously inverted for 16–20 h at 37° C to maximize interactions between ZnO NPs and *S. aureus*. Microcentrifuge tubes containing the same concentrations of ZnO NPs in 400 μL MHB without *S. aureus* were co-incubated at the same time. After incubation, the microcentrifuge tubes were centrifuged at 100 rpm for 2 min to pellet the majority of the ZnO NPs but not *S. aureus*. 200 μL of supernatant was then transferred into a 96-well plate and the OD_600_ values were measured using a Fluostar Omega plate reader (BMG Labtech). The background turbidity from residual ZnO NPs was removed by subtracting the OD_600_ readings for the ZnO control tubes from the tubes containing *S. aureus* and the same concentration of ZnO NPs. For MIC assays using H_2_O_2_, daptomycin, ZnCl_2_, or MgCl_2_, the assays were performed in 96-well plates in a final volume of 200 μL and incubated under static conditions for 16–20 h. For daptomycin assays, MHB was supplemented with 50 μg/mL CaCl_2_. To control for background turbidity caused by the salts (MgCl_2_ and ZnCl_2_), the OD_600_ of wells with the same concentration of salt without *S. aureus* were subtracted from the OD_600_ readings from the wells containing the same concentration of salt with *S. aureus*.

### ZnO NP conditioned media

Conditioned media was made by incubating 20 mg/mL of ZnO NPs in sterile MHB in clear conical tubes at room temperature. At indicated times, the media was mixed to resuspend the ZnO NPs and a 1 mL aliquot of the media was removed and centrifuged at 16,000 rcf for 5 min to pellet the ZnO NPs. 100 μL of the supernatant from this aliquot was then added to an individual well of a 96-well plate. Log phase *S. aureus* was then diluted 1:100 in unconditioned MHB and 100 μL was added to the same wells for a final concentration of 50% conditioned media and approximately 1×10^6^ cfu/ml *S. aureus* in a total volume of 200 μL. As a positive control, *S. aureus* was also grown in unconditioned MHB and as a negative control, 200 μL of conditioned media (no *S. aureus*) was placed in another well. The plate was incubated under static conditions at 37° C and OD_600_ values were measured after 16–20 h. To generate a dose curve of conditioned media, log phase *S. aureus* was diluted 1:100 and 100 μL was added to each well. Then 100 μL of media containing different ratios of conditioned: unconditioned media was added for a total volume of 200 μL containing approximately 1×10^6^ cfu/ml *S. aureus* in a final concentration of 50, 25, and 12.5% conditioned media. Plates were then incubated under static conditions at 37°C for 16–20 h before reading the OD_600_ values.

Chelated media was made by adding 30 mg/mL of Chelex beads (Sigma Aldrich) to the media (either unconditioned MHB or conditioned MHB) and inverting continuously for 1 hour at room temperature. The mixture was then sterile filtered to remove the beads and the process was repeated. Cultures were grown to log phase (OD_600_ of 0.4) and separated into four aliquots. Each of these aliquoted cultures were pelleted and washed in PBS two times before being resuspended in the equivalent volume of either MHB, conditioned media, chelated media, or chelated conditioned media. These aliquots were then diluted 1:50 in their respective media (MHB, chelated MHB, conditioned MHB, or chelated conditioned MHB). 50 μL of the 1:50 cultures was then added to a 96-well plate containing 150 μL of the matching media type to produce a 1:200 final dilution (approximately 1×10^6^ cfu/ml) of *S. aureus* in 100% of each media type. To produce a mix of 25% regular MHB and 75% of each media type (chelated MHB, conditioned MHB, or chelated conditioned MHB), 50 μL of the *S. aureus* culture diluted 1:50 in MHB was added to a 96-well plate containing 150 μL of either chelated media, conditioned media, or chelated conditioned media. The 96-well plate was incubated under static conditions at 37° C and OD_600_ values were measured after 16–20 h.

The concentration of Zn^2+^ in conditioned media was measured by ICP-MS at the University of Texas at Dallas Chemistry Core Facility. MHB was conditioned with 20 mg/mL ZnO NPs for either one-day or at least one-month. An aliquot of the conditioned media was then chelated as described above. 100 μL of each sample was diluted with 4,900 μL of blank for analysis and a 50x correction factor applied to the data. Two independent samples were measured for each condition and reported as mean ± standard deviation.

### Contact-independent ZnO assays (dialysis button)

Empty dialysis buttons (Hampton Research), elastic bands, and 3.5 MWCO semipermeable dialysis tubing (ThermoFisher) were sterilized by UV radiation (254 nm) for at least 15 min on all sides prior to assay set up. Either 0 mg or 30 mg of ZnO NPs was loaded into sterilized dialysis buttons and sterile MHB was added until the button was filled. The dialysis button was then sealed with dialysis tubing soaked in sterile water and secured with elastic bands. Excess tubing was cut to size with disinfected scissors. Log phase *S. aureus* Newman (approximately 2×10^6^ cfu/ml) was grown in the presence of an empty dialysis button, 30 mg free ZnO (no button), or 30 mg of ZnO inside a dialysis button in a total volume of 1.5 mL MHB. To ensure proper sterilization of the dialysis buttons and aseptic technique, an empty dialysis button was incubated in MHB in the absence of bacteria and served as the negative control. To control for background turbidity of free ZnO NPs, 30 mg of ZnO NPs (no button) was incubated in the absence of *S. aureus* in 1.5 mL of MHB. The assays were conducted in individual wells of a 24-well tissue culture plate that was incubated at 37°C shaking at 200 rpm for 16–20 h. After 16–20 h, OD_600_ values were measured. To minimize background from residual ZnO particles, the OD_600_ of free ZnO NPs incubated in the absence of *S. aureus* was subtracted from the OD_600_ of free ZnO NPs incubated with *S. aureus.*

### Statistics

GraphPad Prism (San Diego, CA, USA) was used for all statistical analysis. Statistical significance set as *p*<0.05 unless otherwise indicated.

## Results

### Neither production of H_2_O_2_ nor electrostatic interactions are responsible for ZnO NP-mediated toxicity

**Figure 1:**
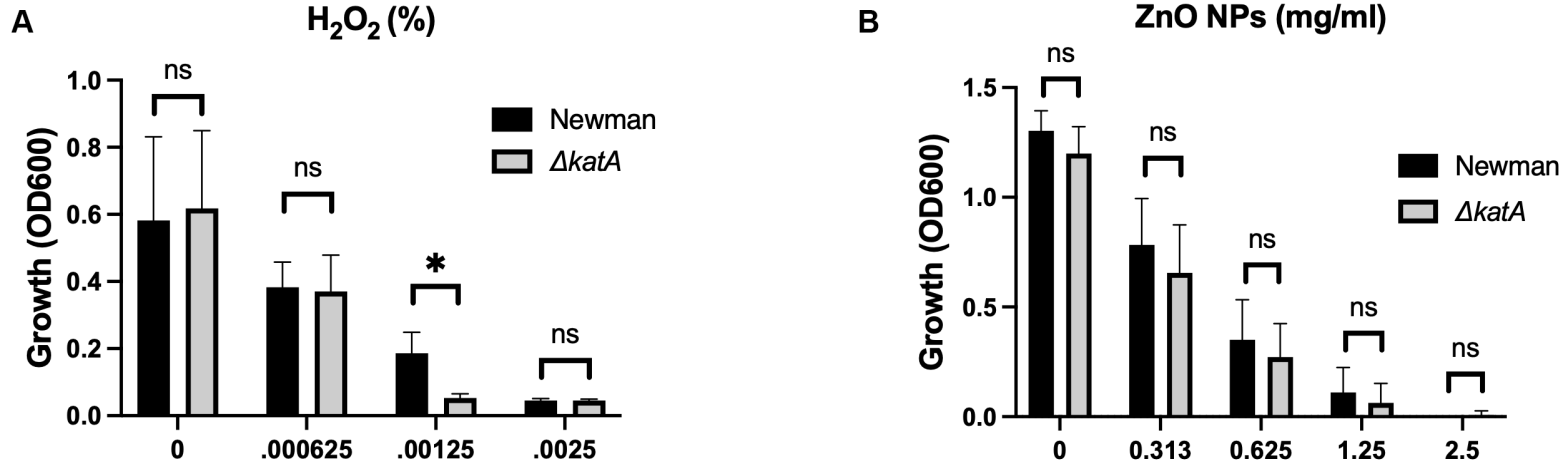
Production of H_2_O_2_ is not responsible for the antimicrobial activity of ZnO NPs. Parental (Newman) and Δ*katA S. aureus* growth in **(A)** H_2_O_2_ and **(B)** ZnO NPs. Data are presented as mean +/− SD of at least three independent trials. Statistical significance determined by unpaired t-test; *p < 0.05 or non-significant (ns) from parental levels within each treatment group.

Several previous studies have demonstrated the antimicrobial activity of ZnO NPs against *S. aureus* using a variety of ZnO NP sources and assay conditions (Raghupathi et al., 2011; Kadiyala et al., 2018; Dadi et al., 2019; de Lucas-Gil et al., 2019; Ali et al., 2021). The ZnO NPs used in this study were purchased from Sigma-Aldrich and no treatment was done on them prior to experimentation. The certificates of analysis from the two lots used have nearly identical parameters including a complexometric titration of 79.7 percent zinc (lot MKBD9523) or 79.8 percent zinc (lot MKCG5504) and an average particle size of 67 nm and specific surface area of 16 m^2^/g (both lots). We used SEM to measure size distribution and shape and found a wide range of sizes with an average of 52 nm (lot MKCG5504) and 48 nm (lot MKBD9523), but with a standard deviation of 39 and 46 nm, respectively, and a granular/mixed shape (Supplementary Figure S1). Despite the size range, our measurements align with the reported values and particle size is less than 100 nm. One challenge in assay setup is that ZnO NPs have low solubility and do not stay suspended in solution. If added to the well of a plate, particularly under static conditions, they will accumulate at the bottom, potentially creating unequal interactions between the ZnO NPs and bacteria in the well. In order to address this, we utilized a ZnO NP assay with continuous inversion that we had previously established (Johnson et al., 2022) as we found better mixing with the ZnO NPs than orbital shaking in a well plate. As seen before, this resulted in inhibition of bacterial growth at an MIC of 1.25 mg/mL (15.4 mM) for ZnO NPs obtained from Sigma-Aldrich (Figure 1B).

Production of H_2_O_2_ by ZnO NPs has historically been the predominant theory proposed for the antibacterial properties of ZnO (Xu et al., 2013; Sirelkhatim et al., 2015; Siddiqi et al., 2018; Tiwari et al., 2018; Lallo Da Silva et al., 2019; Gudkov et al., 2021). The NP surface acts as a catalyst surface to initiate a cascade of oxidation reactions that results in the production of H_2_O_2_. This cascade begins as dissolved oxygen molecules are transformed into superoxide radical anions (O_2_^−^). The O_2_^−^ molecules then react with H^+^ to form HO_2_ which can pick up electrons and become hydroxyl peroxide anions (HO_2_^−^). HO_2_^−^ is inherently unstable and will react with H^+^ to form H_2_O_2_ (Lallo Da Silva et al., 2019; Li et al., 2020). In addition to being more stable than the intermediates, H_2_O_2_ is also neutrally charged and can diffuse through the bacterial membrane to cause intercellular damage (Lallo Da Silva et al., 2019; Li et al., 2020). To evaluate the role of H_2_O_2_ in ZnO NP toxicity, we used a strain of *S. aureus* where the catalase gene (*katA*) was removed via allelic replacement to generate Δ*katA* (Park et al., 2008). Catalase is responsible for breaking down H_2_O_2_ into H_2_O and O_2_ and loss of the gene results in increased susceptibility to H_2_O_2_ (Park et al., 2008). We hypothesized that if H_2_O_2_ is responsible for ZnO-mediated growth inhibition, then Δ*katA* should be more susceptible to ZnO NPs than its parental strain (Newman). We confirmed that Δ*katA* had increased susceptibility to H_2_O_2_ (Figure 1A), but we saw no difference in ZnO NP susceptibility as compared to the parental strain (Figure 1B) indicating that H_2_O_2_ is not responsible for ZnO-mediated toxicity.

**Figure 2:**
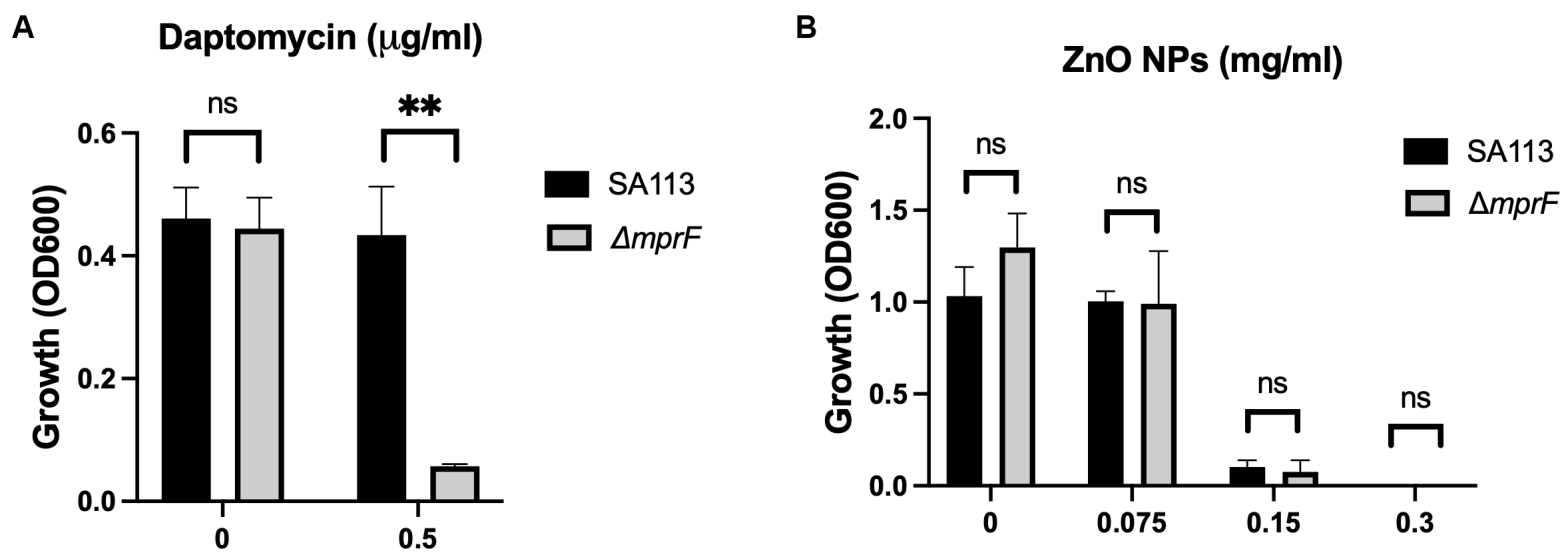
Electrostatic interactions with the bacterial membrane do not mediate ZnO NP toxicity. Parental (SA113) and Δ*mprF S. aureus* growth in **(A)** daptomycin and **(B)** ZnO NPs. Data are presented as mean +/− SD of at least three independent trials. Statistical significance determined by unpaired *t*-test; *p < 0.05 or non-significant (ns) from parental levels within each treatment group.

Physical contact that disrupts cellular integrity is another commonly proposed mechanism of ZnO toxicity. Previous studies have seen evidence of physical interactions between ZnO NPs and the bacterial cell surface and reported damage to the bacterial cell membrane after exposure to ZnO NPs (Feris et al., 2010; Sirelkhatim et al., 2015; Joe et al., 2017; Siddiqi et al., 2018; Akbar et al., 2019; Lallo Da Silva et al., 2019; Gudkov et al., 2021). Additionally, several studies have specifically implicated electrostatic interactions in mediating this contact (Feris et al., 2010; Arakha et al., 2015). To investigate the role of electrostatic interactions in *S. aureus*, we used a *mprF* deficient strain of *S. aureus* (*ΔmprF*) (Peschel et al., 2001). MprF is responsible for synthesizing and translocating lysyl-phosphatidylglycerol, a positively charged phospholipid, to the cell membrane. Loss of *mprF* results in a cell envelope that is more negatively charged compared to the parental strain resulting in increased susceptibility to cationic antimicrobial peptides such as defensins and the antibiotic daptomycin (Peschel et al., 2001; Ernst et al., 2009). We hypothesized that if electrostatic interactions on the bacterial envelope mediate the antibacterial activity of ZnO NPs, *ΔmprF* would exhibit increased susceptibility to ZnO NPs compared to the SA113 parental strain. We confirmed that *ΔmprF* showed increased susceptibility to daptomycin relative to the parental SA113 strain (Figure 2A) but found that there was no difference in susceptibility with ZnO NPs (Figure 2B) under our assay conditions.

### Physical contact is not necessary for ZnO-mediated growth inhibition

**Figure 3:**
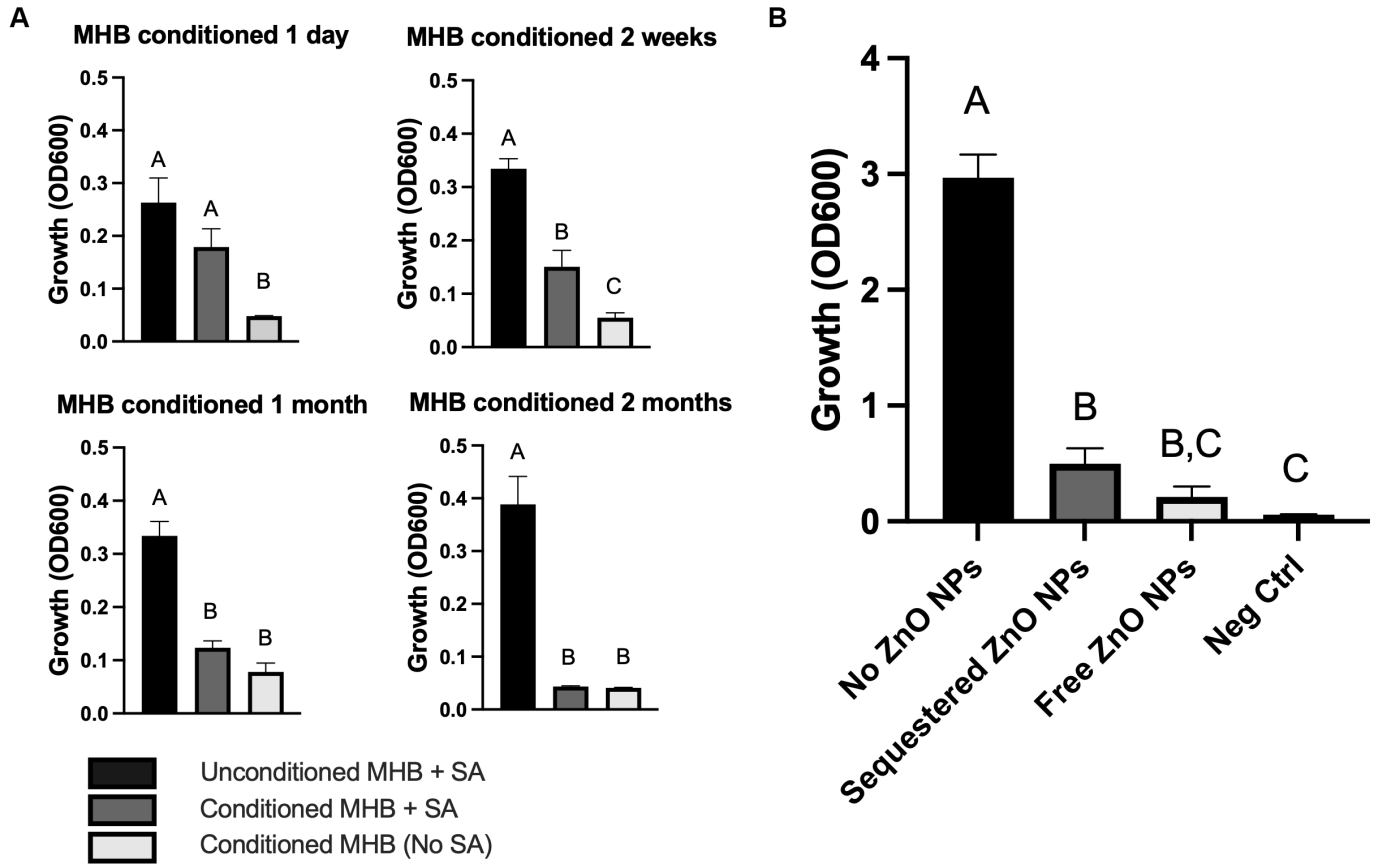
ZnO-mediated growth inhibition is not dependent on physical contact. **(A)** *S. aureus* (SA) Newman growth in unconditioned MHB or MHB conditioned with ZnO NPs for the indicated amounts of time. **(B)** *S. aureus* Newman growth in MHB containing no ZnO NPs, free ZnO NPs, or ZnO NPs sequestered by a dialysis button. The negative control (Neg Ctrl) represents an empty dialysis button incubated in the absence of *S. aureus*. Data are presented as mean +/− SD of at least three independent trials. Statistically significant differences are represented by different letters as determined by a one-way ANOVA followed by Tukey’s *post hoc* test.

Although electrostatic interactions between bacterial and ZnO NP surfaces did not mediate toxicity, it is possible that physical contact and disruption of the membrane occur independent of electrostatic interactions. To evaluate the necessity of physical contact, we conditioned the media by incubating ZnO NPs in MHB in clear conical tubes for up to 2 months, allowing any soluble factors produced by ZnO NPs to accumulate in the media. ZnO NPs were then removed via centrifugation at the indicated times and the ZnO-free, conditioned supernatant was evaluated for the ability to inhibit bacterial growth (Figure 3A). No statistically significant difference in *S. aureus* growth was seen between unconditioned MHB and conditioned MHB after 1 day of conditioning (Figure 3A, upper left panel), but by 2 weeks the ZnO NP-conditioned media exhibited partial growth inhibition (Figure 3A, upper right panel). At one-month, only small amounts of growth were seen in conditioned media although it was not significantly different than the negative control lacking *S. aureus* (Figure 3A, lower left panel). By 2 months, no growth was seen when *S. aureus* was added to conditioned media (Figure 3A, lower right panel). This suggests that ZnO NPs release a soluble species in a time-dependent manner that contributes to bacterial growth inhibition.

We next wanted to investigate whether physical contact was necessary in a real-time assay as it is possible that physical interactions are more critical for toxicity when time is limited (i.e., there is not a month to generate sufficient soluble species for growth inhibition). To do this, we sequestered ZnO NPs inside a dialysis button covered with dialysis tubing which created a semi-permeable membrane that allowed for diffusion of small soluble species across the membrane but prevented any physical contact between the ZnO NPs and bacteria. To ensure that ZnO NPs were unable to diffuse through the dialysis tubing, sequestered ZnO NPs were first incubated in MHB in the absence of bacteria. We found no change in turbidity relative to MHB alone indicating the ZnO NPs remained inside the dialysis membrane (data not shown). We next added *S. aureus* to the assay and found that the sequestered ZnO NPs effectively inhibited bacterial growth relative to the *S. aureus* grown in the absence of any ZnO NPs (Figure 3B). This confirmed that ZnO NP could inhibit bacterial growth in the absence of physical interaction with the cell. To compare the effectiveness of growth inhibition in the absence and presence of physical contact, *S. aureus* was also incubated with free (non-sequestered) ZnO NPs under the same conditions. We found that while there was no statistical difference in *S. aureus* growth between sequestered and free ZnO NPs, only the growth inhibition with the free ZnO NPs showed no statistical difference from our negative control (MHB containing an empty dialysis button but no *S. aureus*). Therefore, while physical contact with ZnO NPs is not required for growth inhibition in our assay, it may still contribute to the effectiveness of the particles.

### Zn^2+^ cations are responsible for ZnO NPs toxicity

**Figure 4:**
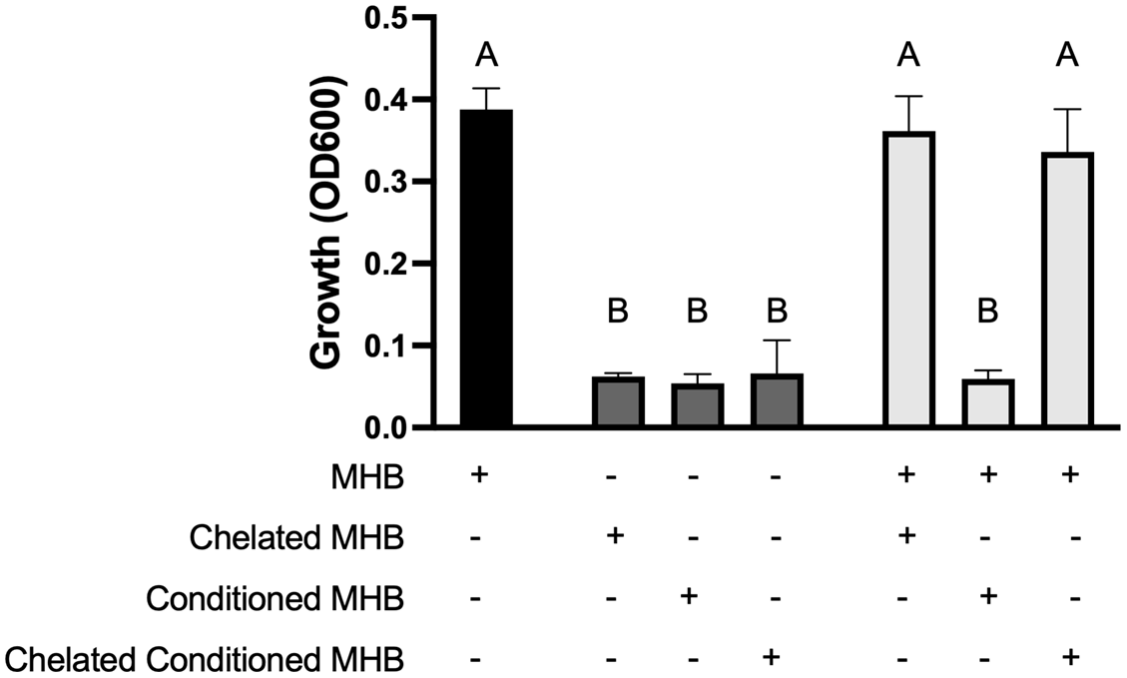
Zn^2+^ ions mediate toxicity. Growth of *S. aureus* Newman in 100% MHB (black bar) or 100% chelated MHB, 100% conditioned media, or 100% chelated conditioned media (dark grey bars) as indicated by the plus (+) sign in the chart. Growth of *S. aureus* in a mixture of 25% MHB and either 75% chelated MHB, 75% conditioned MHB, or 75% chelated conditioned MHB (light grey bars) as indicated by the two plus (+) signs in the chart. Data are presented as mean +/− SD of at least three independent trials. Statistically significant differences are represented by different letters as determined by a one-way ANOVA followed by Tukey’s *post hoc* test.

Although we saw no difference in growth between the parental *S. aureus* and Δ*katA* in the presence of ZnO-NPs (Figure 1B), we wanted to further confirm that H_2_O_2_ is not the soluble species by incubating ZnO NP-conditioned media with the catalase-deficient *S. aureus* stain. Unsurprisingly, we saw no difference in growth between the parental *S. aureus* and Δ*katA* in the presence of conditioned media (data not shown). Previous studies have shown dissolution of Zn^2+^ ions from ZnO NP surfaces that have been linked to microbial toxicity (Pasquet et al., 2014; Sirelkhatim et al., 2015; Siddiqi et al., 2018; de Lucas-Gil et al., 2019; Lallo Da Silva et al., 2019; Song et al., 2020; Gudkov et al., 2021). To assess whether accumulation of Zn^2+^ ions in the conditioned media is the soluble factor responsible for growth inhibition, we used Chelex beads to strip the conditioned media of all divalent cations. *S. aureus* grew well in unconditioned MHB (Figure 4, black bar) but the removal of all divalent cations in chelated unconditioned MHB completely inhibited growth (Figure 4, left dark gray bar). This was expected since the presence of some divalent ions is necessary for bacterial growth. As additional negative controls, we incubated *S. aureus* in ZnO NP-conditioned MHB (Figure 4, middle dark gray bar) and in chelated conditioned MHB (Figure 4, right dark gray bar) and no growth was seen in either condition. We next supplemented our experiments with a small amount (25% of total volume) of regular (non-chelated, unconditioned) MHB to restore enough divalent cations to support the growth of *S. aureus* in the media. When we supplemented the chelated MHB with regular MHB (Figure 4, left light gray bar), it resulted in growth that was no different than our positive control (Figure 4, black bar). However, supplementing with regular MHB was not sufficient for growth when conditioned MHB was included in place of chelated MHB (Figure 4, middle light gray bar) suggesting the presence of a toxic, soluble species only present in conditioned media. Chelating conditioned MHB completely removed this toxic substance resulting once more in full growth of *S. aureus* (Figure 4, right light gray bar) that was no different from our positive control (black bar). Removal of the toxic soluble species through chelation indicates that the toxicity is mediated by divalent cations.

**Figure 5:**
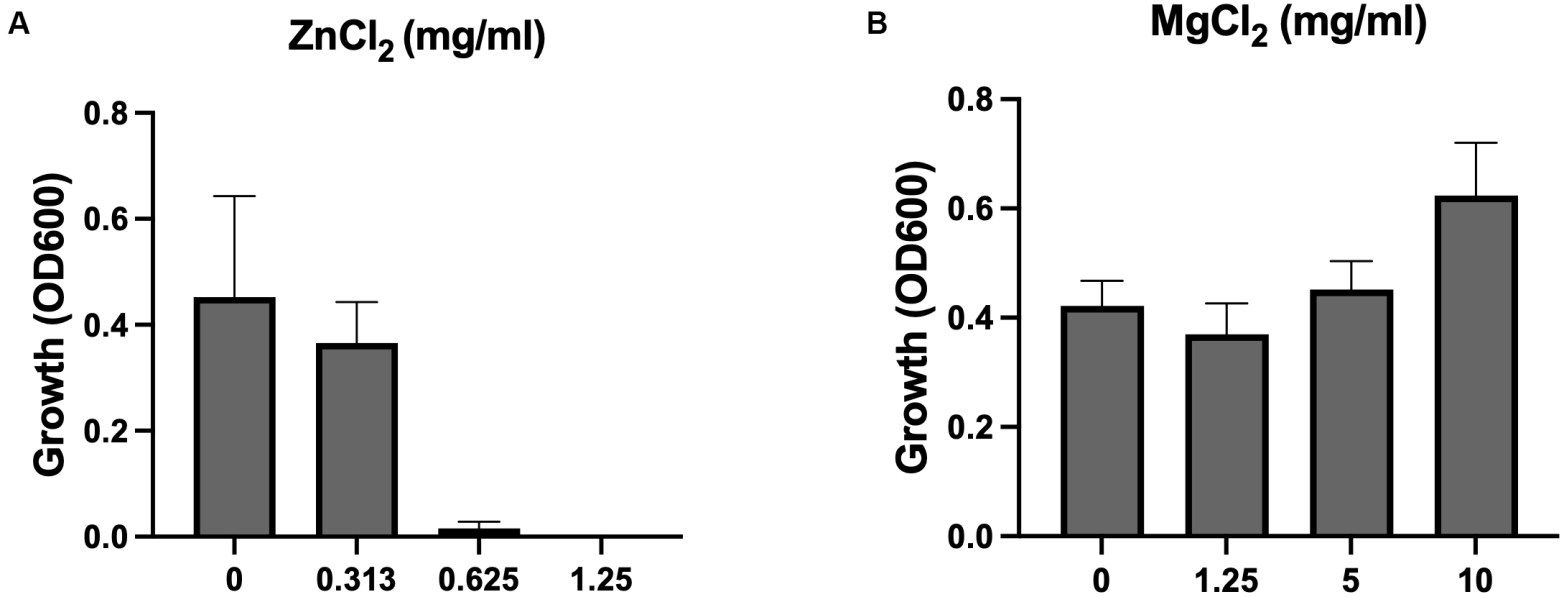
ZnCl_2_ but not MgCl_2_ inhibits growth. Growth of *S. aureus* Newman in **(A)** ZnCl_2_ and **(B)** MgCl_2_. Data are presented as mean +/− SD of at least three independent trials.

To confirm that Zn^2+^ concentration is increased when MHB is conditioned with ZnO NPs, we measured its concentration using ICP-MS. We found that Zn^2+^ concentration increased 100x after one-day of conditioning (31 μM to 3,249 μM) and 300x after at least 1 month of conditioning (31 uM to 9,588 μM) and that this was reversed after chelation (Table 1). We next evaluated susceptibility of *S. aureus* Newman to Zn^2+^ and found that the MIC of ZnCl_2_ was 0.625 mg/mL (4,590 μM) (Figure 5A). It is therefore not surprising that using conditioned media containing Zn^2+^ at a similar concentration would inhibit growth. To determine whether this was the result of an overabundance of divalent cations or if it was specific to Zn^2+^, we conducted an MIC with MgCl_2_ and saw no inhibition of growth up to 10 mg/mL (Figure 5B). Together this suggests that it is the accumulation of Zn^2+^ ions released from ZnO NP surfaces that are mediating the antibacterial mechanism of ZnO NPs in MHB.

**Table 1:** Zn^2+^ concentration in media (mean ± SD).

Media conditions	Zn^2+^ concentration (μM)
MHB	30.8 ± 7.6
MHB conditioned 1 day	3248.9 ± 113.4
MHB conditioned ≥1 month	9588.1 ± 1,256
MHB conditioned ≥1 month and chelated	15.9 ± 19.5

**Figure 6:**
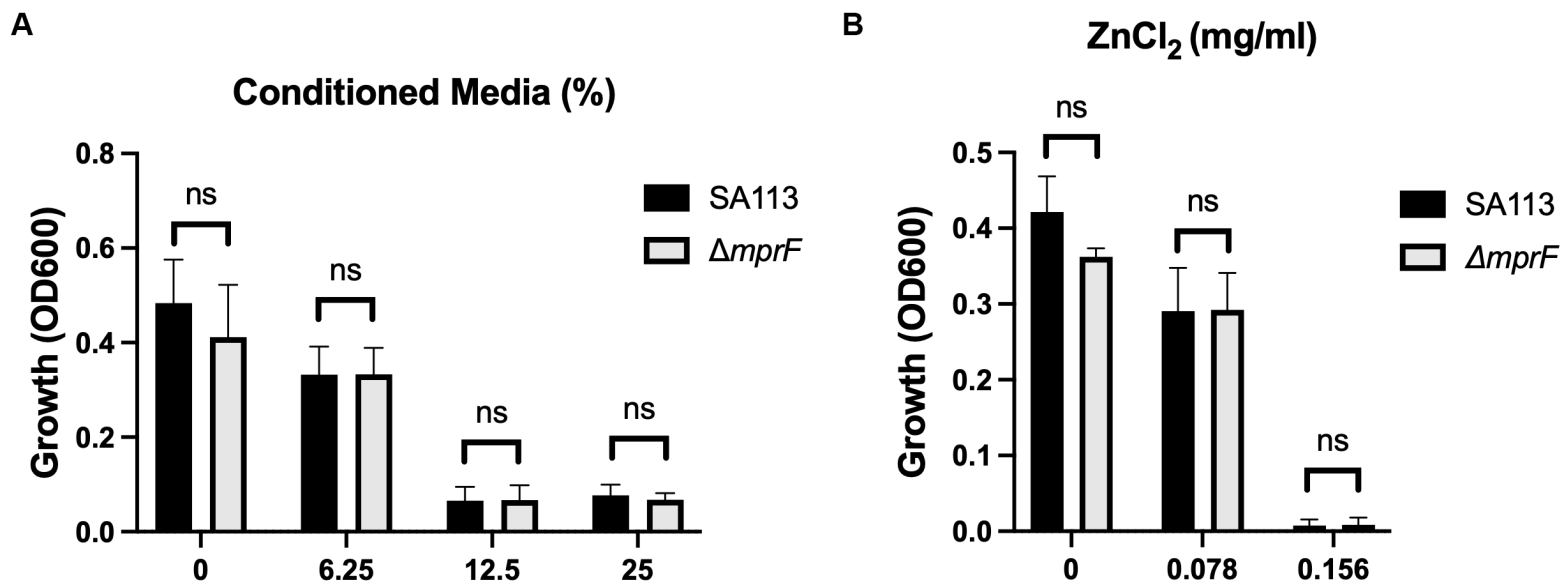
Electrostatic interactions with the bacterial membrane do not mediate Zn^2+^ toxicity. Parental SA113 and Δ*mprF S. aureus* growth in **(A)** conditioned media, and **(B)** ZnCl_2_. Data are presented as mean +/− SD of at least three independent trials. Statistical significance was determined by unpaired *t*-test; non-significant (ns) from parental levels within each treatment group.

Now that we had identified the soluble species as Zn^2+^, we wanted to again look at the potential impact of electrostatic changes to the bacterial membrane using the Δ*mprF* mutant. However, we saw no difference with either our conditioned media (Figure 6A) or Zn^2+^ generated from ZnCl_2_ (Figure 6B). Therefore, while electrostatic interactions may still play a role, they are distinct from what is observed with cationic molecules such as antimicrobial peptides or daptomycin and changes in cell membrane surface charge are not sufficient to effect changes in bacterial susceptibility to Zn^2+^.

## Discussion

Production of ROS, particularly H_2_O_2_, has long been a prevailing theory of ZnO NP-mediated toxicity with multiple studies directly measuring ROS production by ZnO NPs (Xu et al., 2013; Tiwari et al., 2018; Liu et al., 2019; Soltanian et al., 2021). The bigger question is the biological significance of this ROS production. Some studies have seen evidence of lipid peroxidation, which they have attributed to ZnO-mediated ROS generation (Eid et al., 2023), although other studies report an absence of lipid peroxidation (Kadiyala et al., 2018). Further confounding the role of ROS, several studies have reported no increase in the expression of oxidative stress genes in response to exposure to ZnO NPs (Raghupathi et al., 2011; Pati et al., 2014; Kadiyala et al., 2018). We used *S. aureus* with a deletion in the catalase gene, *katA*, to directly examine whether H_2_O_2_ production by ZnO NPs mediates toxicity. Loss of *katA* significantly increases *S. aureus* susceptibility to H_2_O_2_ ((Park et al., 2008) and Figure 1A), yet no change in growth was seen when Δ*katA* was exposed to ZnO relative to the parental strain (Figure 1B). These findings align with gene expression studies that found that ZnO NPs do not induce *katA* expression in *S. aureus* (Raghupathi et al., 2011; Pati et al., 2014; Kadiyala et al., 2018). Although it is possible that ROS other than H_2_O_2_ contribute to toxicity, Kadiyala et al. showed that the powerful antioxidant N-acetylcysteine provided no protective effects against ZnO-NPs (Kadiyala et al., 2018), which provides some evidence that our findings may extend beyond H_2_O_2_. We will note that our results contradict those of Borda et al. who found a correlation between H_2_O_2_ and ZnO NP susceptibility using bacterial species with differing H_2_O_2_ sensitivities (Borda d’ Água et al., 2018). However, this approach cannot control for other variables that would differ between species beyond that of H_2_O_2_ susceptibility. A benefit of using an isogenic mutant is that the only difference between the strains is loss of the *katA* gene and thus any changes in susceptibility can be directly attributable to H_2_O_2_. Although we saw no evidence of H_2_O_2_ involvement in ZnO NP toxicity in our assay conditions, properties of ZnO NPs can differ widely depending on their method of synthesis, physical characteristics, and assay conditions including media type or the presence or absence of light (Li et al., 2011; Xu et al., 2013; de Lucas-Gil et al., 2019). Therefore, while we cannot rule out the role of H_2_O_2_ in mediating ZnO-toxicity under any condition, it is not a universal mechanism.

Physical contact and/or internalization are additional mechanisms attributed to ZnO NPs (Sirelkhatim et al., 2015; Lallo Da Silva et al., 2019). We previously investigated the role of internalization in ZnO mediated toxicity using microparticles incapable of being internalized and found that ZnO still inhibited *S. aureus* growth (Reeks et al., 2021). However, this does not rule out the necessity of physical contact in disruption of the cell envelope. Membrane damage after contact with ZnO NPs has been reported using SEM or TEM images (Liu et al., 2009; Manzoor et al., 2016; Joe et al., 2017; Siddiqi et al., 2018; Akbar et al., 2019; Reeks et al., 2021; Dhanasegaran et al., 2022; Johnson et al., 2022). Several means of interaction have been proposed including the role of proteins and polysaccharides mediating attachment (Dhanasegaran et al., 2022) as well as electrostatic interactions (Feris et al., 2010; Arakha et al., 2015). Although we saw no evidence of electrostatic interactions with ZnO NPs (Figure 2B), previous studies concluded that ZnO NPs with positive surface potentials have higher toxicity than synonymous ZnO NPs with negative surface potentials (Feris et al., 2010; Arakha et al., 2015). The ZnO NPs used in our assays have a negative net surface potential as measured by Zeta potential measurements (data not shown) and therefore it is possible that electrostatic interactions could be seen with ZnO NPs having a more positive surface potential. Nonetheless, our results with ZnCl_2_ indicate that changes in cell membrane surface charge is not sufficient to effect changes in bacterial susceptibility to Zn^2+^ itself (Figure 6B).

Despite the evidence of membrane damage after ZnO NP exposure (Akbar et al., 2019; Dhanasegaran et al., 2022), our results indicate that physical contact may not be strictly necessary for ZnO-mediated toxicity. Few studies have evaluated ZnO cytotoxicity in the absence of physical contact and it is possible that damage to the cell membrane may occur through a mechanism independent of physical contact. We found that soluble Zn^2+^ accumulates in MHB over time (Table 1) leading to bacterial growth inhibition (Figure 3A) and that this toxicity was reversed by removal of Zn^2+^ (Figure 4). This inhibition was also seen in real-time when ZnO NPs were physically separated from bacterial cells by a membrane permeable to soluble species but not the particles themselves (Figure 3B). However, although we saw significant inhibition with sequestered ZnO NPs, there was still more growth relative to the negative control than with free (non-sequestered) ZnO NPs. Therefore, while physical contact may not be necessary, it could still contribute to toxicity.

Our results point to Zn^2+^ ions, rather than H_2_O_2_, as the soluble species mediating ZnO NP toxicity. Zn^2+^ toxicity may occur through two mechanisms: (1) mis-metalation of enzymes that require metal cofactors leading to protein dysfunction and (2) disruption of osmotic homeostasis (Lemire et al., 2013; Sirelkhatim et al., 2015; Siddiqi et al., 2018; de Lucas-Gil et al., 2019; Song et al., 2020; Gudkov et al., 2021). While the exact role of Zn^2+^ in cytotoxicity is still unconfirmed, the ability of Zn^2+^ ions to induce bacterial death has been established (McDevitt et al., 2011; Lemire et al., 2013). Despite this, there is relatively little known about the molecular targets. McDevitt et al. reports that Zn^2+^ inhibits acquisition of Mn^2+^ by competing for binding to the solute binding protein PsaA, causing Mn^2+^ starvation, growth inhibition, and susceptibility to oxidative stress (McDevitt et al., 2011). Enzymes that contain sulfhydryl moieties are also likely to be the biological targets of soft metals such as zinc (Xu and Imlay, 2012). These moieties are commonly found in dehydratase-family enzymes that are directly involved in catalysis of key metabolic pathways, and their inactivation can arrest growth. However, Zn^2+^ could potentially competitively bind with many different metal binding moieties as 30 to 50% of proteins are predicted to be dependent on metal atoms for their structure and function (Andreini et al., 2004).

Zn^2+^ dissolution is highly dependent on both the ZnO NP surface as well as interactions with the media (Li et al., 2011; Herrmann et al., 2014; Pasquet et al., 2014; Joe et al., 2017; Johnson et al., 2022). Several studies have shown that Zn^2+^ dissolution occurs at a higher rate in media (nutrient broth, Luria-Bertani, or MHB) than in water and they speculate that the presence of proteins or other ionic components are increasing the solubility of ZnO (Li et al., 2011; Pasquet et al., 2014; Joe et al., 2017). Several groups have also found that soluble components such as phosphates, Na^+^, Fe^+^, and some organic molecules alter both the release profiles and the toxicity of Zn^2+^ after its release (Li et al., 2011; Herrmann et al., 2014; Pasquet et al., 2014; Song et al., 2020; Johnson et al., 2022). The presence and nature of surface defects, which are tied to various particle properties such as size, morphology and synthesis conditions, also influences the ability of ZnO particles to release Zn^2+^ (de Lucas-Gil et al., 2019; Song et al., 2020; Johnson et al., 2022). We previously found that there exist relevant morphological differences in the form of an increased abundance of surface trap states related to oxygen deficiencies at the polar surfaces of ZnO microparticles, which indicates an excess of Zn^2+^ ions at these surfaces that can be released into solutions (Johnson et al., 2022). In light of this, it may be possible to modify ZnO NPs to enhance their bacterial growth inhibition. This can be done during synthesis via alternate synthesis methods (Ralphs et al., 2013; Xia et al., 2014; Su et al., 2017), conditions (Xing et al., 2010; Asok et al., 2012), precursors (Wang et al., 2012; Guo et al., 2015), or the addition of dopants (Gao et al., 2016; Lakshmi and Vijayaraghavan, 2017) all of which can impart desirable morphologies and chemical properties. Post synthesis treatment is also a viable method for improving the antibacterial activity as annealing in anaerobic environments and surface functionalization can modify the surface defect structure potentially leading to increased Zn^2+^ release (Cui et al., 2012; Lv et al., 2013; Laurenti et al., 2017; Elhaj Baddar et al., 2019).

A challenge in studying ZnO NP-mediated bacterial toxicity is the extensive variability in particle morphology, media type, and even experimental conditions such as the presence or absence of light, all of which can interact and potentially have profound effects on activity (Li et al., 2011; Xu et al., 2013; Pasquet et al., 2014; Joe et al., 2017; de Lucas-Gil et al., 2019; Song et al., 2020). This demonstrates the need for extensive characterization of the nanoparticles, as well as standardized test conditions when studying the mechanism of ZnO toxicity. It also may explain the contradictory findings regarding the production of ROS, dissolution of Zn^2+^, and necessity of physical contact in ZnO NP-mediated toxicity. Our own assays were all performed in MHB largely because MHB has historically been the standard media for MIC assays, but it is important to note that our results may be specific to MHB, and the mechanism of action could vary with other media or particle types. Additional work investigating this interplay will be critical for maximizing the antibacterial activity of ZnO NPs and optimizing their use in future applications.

## Data availability statement

The raw data supporting the conclusions of this article will be made available by the authors, without undue reservation.

## Author contributions

AC: Formal analysis, Investigation, Methodology, Writing – original draft, Writing – review & editing. IA: Investigation, Methodology, Writing – review & editing. MD: Investigation, Writing – review & editing. DJ: Investigation, Writing – original draft, Writing – review & editing. JR: Conceptualization, Methodology, Writing – review & editing. YS: Conceptualization, Funding acquisition, Project administration, Supervision, Writing – review & editing. SM: Conceptualization, Formal analysis, Funding acquisition, Methodology, Project administration, Supervision, Writing – original draft, Writing – review & editing.

## References

[ref1] Akbar A. Sadiq M. B. Ali I. Muhammad N. Rehman Z. Khan M. N.. (2019). Synthesis and antimicrobial activity of zinc oxide nanoparticles against foodborne pathogens salmonella typhimurium and *Staphylococcus aureus*. Biocatal. Agric. Biotechnol. 17, 36–42. doi: 10.1016/j.bcab.2018.11.005

[ref2] Ali S. S. Moawad M. S. Hussein M. A. Azab M. Abdelkarim E. A. Badr A.. (2021). Efficacy of metal oxide nanoparticles as novel antimicrobial agents against multi-drug and multi-virulent *Staphylococcus aureus* isolates from retail raw chicken meat and giblets. Int. J. Food Microbiol. 344:109116. doi: 10.1016/j.ijfoodmicro.2021.109116, PMID: 33676332

[ref3] Andreini C. Bertini I. Rosato A. (2004). A hint to search for metalloproteins in gene banks. Bioinformatics 20, 1373–1380. doi: 10.1093/bioinformatics/bth095, PMID: 14962940

[ref4] Arakha M. Saleem M. Mallick B. C. Jha S. (2015). The effects of interfacial potential on antimicrobial propensity of ZnO nanoparticle. Sci. Rep. 5:9578. doi: 10.1038/srep09578, PMID: 25873247 PMC4397836

[ref5] Asok A. Gandhi M. N. Kulkarni A. R. (2012). Enhanced visible photoluminescence in ZnO quantum dots by promotion of oxygen vacancy formation. Nanoscale 4, 4943–4946. doi: 10.1039/C2NR31044A, PMID: 22790095

[ref6] Borda d’ Água R. Branquinho R. Duarte M. P. Maurício E. Fernando A. L. Martins R.. (2018). Efficient coverage of ZnO nanoparticles on cotton fibres for antibacterial finishing using a rapid and low cost in situ synthesis. New J. Chem. 42, 1052–1060. doi: 10.1039/C7NJ03418K

[ref7] Centers for Disease Control. (2019). Antibiotic resistance threats in the United States. Atlanta, GA: U.S.: Department of Health and Human Services, CDC doi: 10.15620/cdc:82532

[ref8] Cheung G. Y. C. Bae J. S. Otto M. (2021). Pathogenicity and virulence of *Staphylococcus aureus*. Virulence 12, 547–569. doi: 10.1080/21505594.2021.1878688, PMID: 33522395 PMC7872022

[ref9] Cui L. Zhang H.-Y. Wang G.-G. Yang F.-X. Kuang X.-P. Sun R.. (2012). Effect of annealing temperature and annealing atmosphere on the structure and optical properties of ZnO thin films on sapphire (0001) substrates by magnetron sputtering. Appl. Surf. Sci. 258, 2479–2485. doi: 10.1016/j.apsusc.2011.10.076

[ref10] Dadi R. Azouani R. Traore M. Mielcarek C. Kanaev A. (2019). Antibacterial activity of ZnO and CuO nanoparticles against gram positive and gram negative strains. Mater. Sci. Eng. C 104:109968. doi: 10.1016/j.msec.2019.109968, PMID: 31500003

[ref11] de Lucas-Gil E. Del Campo A. Pascual L. Monte-Serrano M. Menéndez J. Fernández J. F.. (2019). The fight against multidrug-resistant organisms: the role of ZnO crystalline defects. Mater. Sci. Eng. C 99, 575–581. doi: 10.1016/j.msec.2019.02.004, PMID: 30889732

[ref12] DeLeo F. R. Chambers H. F. (2009). Reemergence of antibiotic-resistant *Staphylococcus aureus* in the genomics era. J. Clin. Invest. 119, 2464–2474. doi: 10.1172/JCI38226, PMID: 19729844 PMC2735934

[ref13] Dhanasegaran K. Djearamane S. Liang S. Ling Shing W. Kasivelu G. Lee P. F.. (2022). Antibacterial properties of zinc oxide nanoparticles on *Pseudomonas aeruginosa* (ATCC 27853). Sci. Iran. 28, 3806–3815. doi: 10.24200/sci.2021.56815.4974

[ref14] Eid A. M. Sayed O. M. Hozayen W. Dishisha T. (2023). Mechanistic study of copper oxide, zinc oxide, cadmium oxide, and silver nanoparticles-mediated toxicity on the probiotic *Lactobacillus reuteri*. Drug Chem. Toxicol. 46, 825–840. doi: 10.1080/01480545.2022.2104865 35930385

[ref15] Elhaj Baddar Z. Matocha C. J. Unrine J. M. (2019). Surface coating effects on the sorption and dissolution of ZnO nanoparticles in soil. Environ. Sci. Nano 6, 2495–2507. doi: 10.1039/C9EN00348G

[ref16] Ernst C. M. Staubitz P. Mishra N. N. Yang S.-J. Hornig G. Kalbacher H.. (2009). The bacterial Defensin resistance protein MprF consists of separable domains for lipid Lysinylation and antimicrobial peptide repulsion. PLoS Pathog. 5:e1000660. doi: 10.1371/journal.ppat.1000660, PMID: 19915718 PMC2774229

[ref17] Feris K. Otto C. Tinker J. Wingett D. Punnoose A. Thurber A.. (2010). Electrostatic interactions affect nanoparticle-mediated toxicity to gram-negative bacterium *Pseudomonas aeruginosa* PAO1. Langmuir 26, 4429–4436. doi: 10.1021/la903491z, PMID: 20000362

[ref18] Fischbach M. A. Walsh C. T. (2009). Antibiotics for emerging pathogens. Science 325, 1089–1093. doi: 10.1126/science.1176667, PMID: 19713519 PMC2802854

[ref19] Gao Q. Dai Y. Li C. Yang L. Li X. Cui C. (2016). Correlation between oxygen vacancies and dopant concentration in Mn-doped ZnO nanoparticles synthesized by co-precipitation technique. J. Alloys Compd. 684, 669–676. doi: 10.1016/j.jallcom.2016.05.227

[ref20] Gudkov S. V. Burmistrov D. E. Serov D. A. Rebezov M. B. Semenova A. A. Lisitsyn A. B. (2021). A Mini review of antibacterial properties of ZnO nanoparticles. Front. Phys. 9:641481. doi: 10.3389/fphy.2021.641481 PMC8300809 34356805

[ref21] Guo H.-L. Zhu Q. Wu X.-L. Jiang Y.-F. Xie X. Xu A.-W. (2015). Oxygen deficient ZnO1−x nanosheets with high visible light photocatalytic activity. Nanoscale 7, 7216–7223. doi: 10.1039/C5NR00271K 25812132

[ref22] Heidary M. Bostanabad S. Z. Amini S. M. Jafari A. Nobar M. G. Ghodousi A.. (2019). The anti-mycobacterial activity of ag, ZnO, and ag-ZnO nanoparticles against MDR -and XDR-*Mycobacterium tuberculosis*. Infect. Drug Resist. 12, 3425–3435. doi: 10.2147/idr.S221408, PMID: 31807033 PMC6839584

[ref23] Herrmann R. García-García F. J. Reller A. (2014). Rapid degradation of zinc oxide nanoparticles by phosphate ions. Beilstein J. Nanotechnol. 5, 2007–2015. doi: 10.3762/bjnano.5.209, PMID: 25383310 PMC4222298

[ref24] Joe A. Park S.-H. Shim K.-D. Kim D.-J. Jhee K.-H. Lee H.-W.. (2017). Antibacterial mechanism of ZnO nanoparticles under dark conditions. J. Ind. Eng. Chem. 45, 430–439. doi: 10.1016/j.jiec.2016.10.013

[ref25] Johnson D. Reeks J. M. Caron A. Tzoka I. Ali I. McGillivray S. M.. (2022). Influence of surface properties and microbial growth media on antibacterial action of ZnO. Coatings 12:1648. doi: 10.3390/coatings12111648

[ref26] Kadiyala U. Turali-Emre E. S. Bahng J. H. Kotov N. A. Vanepps J. S. (2018). Unexpected insights into antibacterial activity of zinc oxide nanoparticles against methicillin resistant *Staphylococcus aureus* (MRSA). Nanoscale 10, 4927–4939. doi: 10.1039/c7nr08499d, PMID: 29480295 PMC5847298

[ref27] Lakshmi P. V. Vijayaraghavan R. (2017). Chemical manipulation of oxygen vacancy and antibacterial activity in ZnO. Mater. Sci. Eng. C 77, 1027–1034. doi: 10.1016/j.msec.2017.03.280, PMID: 28531975

[ref28] Lallo Da Silva B. Abuçafy M. P. Berbel Manaia E. Oshiro Junior J. A. Chiari-Andréo B. G. Pietro R. C. R.. (2019). Relationship between structure and antimicrobial activity of zinc oxide nanoparticles: an overview. Int. J. Nanomedicine 14, 9395–9410. doi: 10.2147/ijn.s216204, PMID: 31819439 PMC6897062

[ref29] Laurenti M. Stassi S. Canavese G. Cauda V. (2017). Surface engineering of nanostructured ZnO surfaces. Adv. Mater. Interfaces 4:1600758. doi: 10.1002/admi.201600758

[ref30] Lemire J. A. Harrison J. J. Turner R. J. (2013). Antimicrobial activity of metals: mechanisms, molecular targets and applications. Nat. Rev. Microbiol. 11, 371–384. doi: 10.1038/nrmicro3028 23669886

[ref31] Li Y. Liao C. Tjong S. C. (2020). Recent advances in zinc oxide nanostructures with antimicrobial activities. Int. J. Mol. Sci. 21:8836. doi: 10.3390/ijms21228836, PMID: 33266476 PMC7700383

[ref32] Li Q. Mahendra S. Lyon D. Y. Brunet L. Liga M. V. Li D.. (2008). Antimicrobial nanomaterials for water disinfection and microbial control: potential applications and implications. Water Res. 42, 4591–4602. doi: 10.1016/j.watres.2008.08.015, PMID: 18804836

[ref33] Li M. Zhu L. Lin D. (2011). Toxicity of ZnO nanoparticles to *Escherichia coli*: mechanism and the influence of medium components. Environ. Sci. Technol. 45, 1977–1983. doi: 10.1021/es102624t, PMID: 21280647

[ref34] Liu Y. He L. Mustapha A. Li H. Hu Z. Q. Lin M. (2009). Antibacterial activities of zinc oxide nanoparticles against *Escherichia coli* O157:H7. J. Appl. Microbiol. 107, 1193–1201. doi: 10.1111/j.1365-2672.2009.04303.x, PMID: 19486396

[ref35] Liu S. Lai Y. Zhao X. Li R. Huang F. Zheng Z.. (2019). The influence of H2O2 on the antibacterial activity of ZnO. Mater. Res. Express 6:0850c0856. doi: 10.1088/2053-1591/ab2506

[ref36] Lv Y. Pan C. Ma X. Zong R. Bai X. Zhu Y. (2013). Production of visible activity and UV performance enhancement of ZnO photocatalyst via vacuum deoxidation. Appl. Catal. B Environ. 138-139, 26–32. doi: 10.1016/j.apcatb.2013.02.011

[ref37] Manzoor U. Siddique S. Ahmed R. Noreen Z. Bokhari H. Ahmad I. (2016). Antibacterial, structural and optical characterization of Mechano-chemically prepared ZnO nanoparticles. PLoS One 11:e0154704. doi: 10.1371/journal.pone.0154704, PMID: 27183165 PMC4868307

[ref38] McDevitt C. A. Ogunniyi A. D. Valkov E. Lawrence M. C. Kobe B. McEwan A. G.. (2011). A molecular mechanism for bacterial susceptibility to zinc. PLoS Pathog. 7:e1002357. doi: 10.1371/journal.ppat.1002357, PMID: 22072971 PMC3207923

[ref39] Modi S. Inwati G. K. Gacem A. Saquib Abullais S. Prajapati R. Yadav V. K.. (2022). Nanostructured antibiotics and their emerging medicinal applications: an overview of Nanoantibiotics. Antibiotics 11:708. doi: 10.3390/antibiotics11060708, PMID: 35740115 PMC9219893

[ref40] Park B. Nizet V. Liu G. Y. (2008). Role of *Staphylococcus aureus* catalase in niche competition against *Streptococcus pneumoniae*. J. Bacteriol. 190, 2275–2278. doi: 10.1128/jb.00006-08, PMID: 18223076 PMC2293205

[ref41] Pasquet J. Chevalier Y. Pelletier J. Couval E. Bouvier D. Bolzinger M.-A. (2014). The contribution of zinc ions to the antimicrobial activity of zinc oxide. Colloids Surf. A Physicochem. Eng. Asp. 457, 263–274. doi: 10.1016/j.colsurfa.2014.05.057

[ref42] Pati R. Mehta R. K. Mohanty S. Padhi A. Sengupta M. Vaseeharan B.. (2014). Topical application of zinc oxide nanoparticles reduces bacterial skin infection in mice and exhibits antibacterial activity by inducing oxidative stress response and cell membrane disintegration in macrophages. Nanomedicine 10, 1195–1208. doi: 10.1016/j.nano.2014.02.012, PMID: 24607937

[ref43] Peschel A. Jack R. W. Otto M. Collins L. V. Staubitz P. Nicholson G.. (2001). *Staphylococcus aureus* resistance to human defensins and evasion of neutrophil killing via the novel virulence factor MprF is based on modification of membrane lipids with l-lysine. J. Exp. Med. 193, 1067–1076. doi: 10.1084/jem.193.9.1067, PMID: 11342591 PMC2193429

[ref44] Preeti R. Radhakrishnan V. S. Mukherjee S. Mukherjee S. Singh S. P. Prasad T. (2020). ZnO quantum dots: broad Spectrum Microbicidal agent against multidrug resistant Pathogens *E. Coli* and *C. albicans*. Front. Nanotechnol. 2:576342. doi: 10.3389/fnano.2020.576342

[ref45] Raghupathi K. R. Koodali R. T. Manna A. C. (2011). Size-dependent bacterial growth inhibition and mechanism of antibacterial activity of zinc oxide nanoparticles. Langmuir 27, 4020–4028. doi: 10.1021/la104825u, PMID: 21401066

[ref46] Ralphs K. Hardacre C. James S. L. (2013). Application of heterogeneous catalysts prepared by mechanochemical synthesis. Chem. Soc. Rev. 42, 7701–7718. doi: 10.1039/C3CS60066A, PMID: 23752744

[ref47] Reeks J. M. Ali I. Moss W. J. Davis E. McGillivray S. M. Strzhemechny Y. M. (2021). Microscale ZnO with controllable crystal morphology as a platform to study antibacterial action on *Staphylococcus aureus*. Biointerphases 16:031003. doi: 10.1116/6.0000957, PMID: 34241188

[ref48] Siddiqi K. S. Ur Rahman A. Tajuddin H. A. (2018). Properties of zinc oxide nanoparticles and their activity against microbes. Nanoscale Res. Lett. 13:141. doi: 10.1186/s11671-018-2532-3, PMID: 29740719 PMC5940970

[ref49] Sirelkhatim A. Mahmud S. Seeni A. Kaus N. H. M. Ann L. C. Bakhori S. K. M.. (2015). Review on zinc oxide nanoparticles: antibacterial activity and toxicity mechanism. Nanomicro. Lett. 7, 219–242. doi: 10.1007/s40820-015-0040-x, PMID: 30464967 PMC6223899

[ref50] Smaoui S. Chérif I. Ben Hlima H. Khan M. U. Rebezov M. Thiruvengadam M.. (2023). Zinc oxide nanoparticles in meat packaging: A systematic review of recent literature. Food Packag. Shelf Life 36:101045. doi: 10.1016/j.fpsl.2023.101045

[ref51] Soltanian S. Sheikhbahaei M. Mohamadi N. Pabarja A. Abadi M. F. S. Tahroudi M. H. M. (2021). Biosynthesis of zinc oxide nanoparticles using Hertia intermedia and evaluation of its cytotoxic and antimicrobial activities. BioNanoScience 11, 245–255. doi: 10.1007/s12668-020-00816-z

[ref52] Song K. Zhang W. Sun C. Hu X. Wang J. Yao L. (2020). Dynamic cytotoxicity of ZnO nanoparticles and bulk particles to *Escherichia coli*: a view from unfixed ZnO particle: Zn2+ ratio. Aquat. Toxicol. 220:105407. doi: 10.1016/j.aquatox.2020.105407, PMID: 31945654

[ref53] Su X.-F. Chen J.-B. He R.-M. Li Y. Wang J. Wang C.-W. (2017). The preparation of oxygen-deficient ZnO nanorod arrays and their enhanced field emission. Mater. Sci. Semicond. Process. 67, 55–61. doi: 10.1016/j.mssp.2017.05.012

[ref54] Tacconelli E. Carrara E. Savoldi A. Harbarth S. Mendelson M. Monnet D. L.. (2018). Discovery, research, and development of new antibiotics: the WHO priority list of antibiotic-resistant bacteria and tuberculosis. Lancet Infect. Dis. 18, 318–327. doi: 10.1016/s1473-3099(17)30753-3, PMID: 29276051

[ref55] Tiwari V. Mishra N. Gadani K. Solanki P. S. Shah N. A. Tiwari M. (2018). Mechanism of anti-bacterial activity of zinc oxide nanoparticle against Carbapenem-resistant *Acinetobacter baumannii*. Front. Microbiol. 9:1218. doi: 10.3389/fmicb.2018.01218, PMID: 29928271 PMC5997932

[ref56] Wang J. Wang Z. Huang B. Ma Y. Liu Y. Qin X.. (2012). Oxygen vacancy induced band-gap narrowing and enhanced visible light photocatalytic activity of ZnO. ACS Appl. Mater. Interfaces 4, 4024–4030. doi: 10.1021/am300835p, PMID: 22786575

[ref9001] World Health Organization. (2017). Prioritization of pathogens to guide discovery, research and development of new antibiotics for drug-resistant bacterial infections, including tuberculosis. Geneva: World Health Organization (WHO/EMP/IAU/2017.12).

[ref57] Xia T. Wallenmeyer P. Anderson A. Murowchick J. Liu L. Chen X. (2014). Hydrogenated black ZnO nanoparticles with enhanced photocatalytic performance. RSC Adv. 4, 41654–41658. doi: 10.1039/C4RA04826A

[ref58] Xing G. Wang D. Yi J. Yang L. Gao M. He M.. (2010). Correlated d ferromagnetism and photoluminescence in undoped ZnO nanowires. Appl. Phys. Lett. 96:112511. doi: 10.1063/1.3340930

[ref59] Xu X. Chen D. Yi Z. Jiang M. Wang L. Zhou Z.. (2013). Antimicrobial mechanism based on H2O2 generation at oxygen vacancies in ZnO crystals. Langmuir 29, 5573–5580. doi: 10.1021/la400378t, PMID: 23570415

[ref60] Xu F. F. Imlay J. A. (2012). Silver(I), mercury(II), cadmium(II), and zinc(II) target exposed enzymic iron-sulfur clusters when they toxify *Escherichia coli*. Appl. Environ. Microbiol. 78, 3614–3621. doi: 10.1128/aem.07368-11, PMID: 22344668 PMC3346352

