# Supplementary material for: Zinc oxide nanoparticles mediate bacterial toxicity in Mueller-Hinton Broth via Zn2+
Source: Front Microbiol. 2024 Apr 22;15:1394078. doi: 10.3389/fmicb.2024.1394078 (PMC11070567; doi:10.3389/fmicb.2024.1394078)
Supplement: Supplementary file 1 [file Data_Sheet_1.PDF]

Supplementary Material

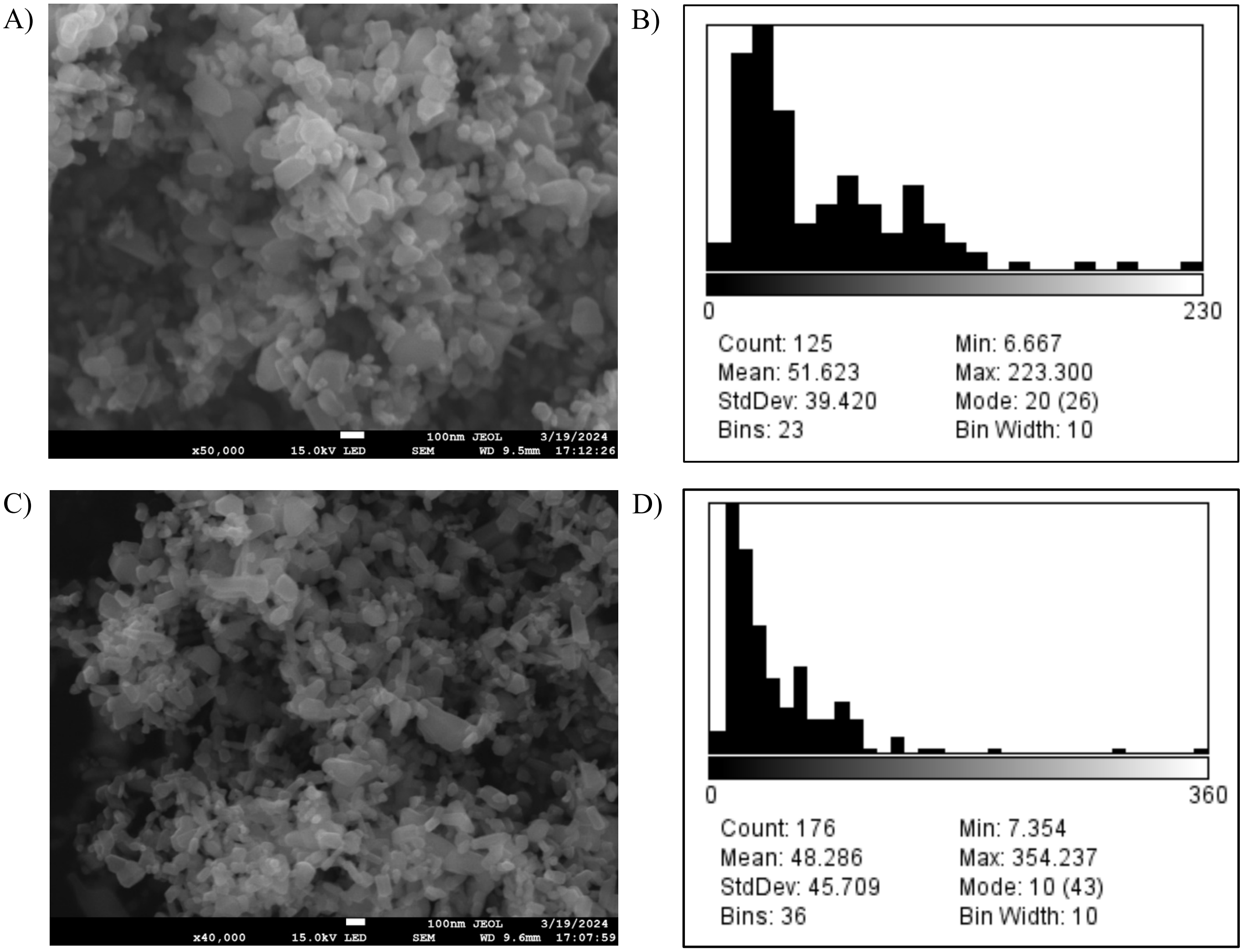

Supplementary Figure 1. SEM images and length measurements of Sigma-Aldrich NPs. Lot MKCG5504 (A) surface morphology and (B) length measurements at x50,000 magnification. Lot MKBD9523 (C) surface morphology and (D) length measurements at x40,000 magnification.
